# Open reduction and internal fixation of displaced proximal humeral fractures. Does the surgeon‘s experience have an impact on outcomes?

**DOI:** 10.1371/journal.pone.0207044

**Published:** 2018-11-06

**Authors:** Tobias Helfen, Georg Siebenbürger, Evi Fleischhacker, Niklas Biermann, Wolfgang Böcker, Ben Ockert

**Affiliations:** Munich University Hospital, Dept. of General, Trauma and Reconstructive Surgery, Ludwig-Maximilians-University, Munich, Germany; Rothman Institute, UNITED STATES

## Abstract

**Introduction:**

To evaluate outcomes following open reduction and internal fixation of displaced proximal humeral fractures with regards to the surgeon’s experience.

**Material and methods:**

Patients were included undergoing ORIF by use of locking plates for displaced two-part surgical neck type proximal humeral fractures. Reduction and functional outcomes were compared between procedures that were conducted by trauma surgeons [TS], senior (>2 years after board certified) trauma surgeons [STS] and trauma surgeons performing ≥50 shoulder surgeries per year [SS]. Quality of reduction was measured on postoperative x-rays. Functional outcomes were assessed by gender- and age-related Constant Score (nCS). Secondary outcome measures were complication and revision rates.

**Results:**

Between 2002–2014 (12.5 years) n = 278 two-part surgical neck type humeral fractures (AO 11-A2, 11-A3) were included. Open reduction and internal fixation was performed with the following educational levels: [TS](n = 68, 25.7%), [STS](n = 110, 41.5%) and [SS](n = 77, 29.1%). Functional outcome (nCS) increased with each higher level of experience and was significantly superior in [SS] (93.3) vs. [TS] (79.6; p = 0.01) vs. [STS] (83.0; p = 0.05). [SS] (7.8%) had significantly less complications compared with [TS] (11.3%; p = 0.003) and [STS](11.7%; p = 0.01) moreover significantly less revision rates (3.9%) vs. [TS](8.2%) and [STS](7.4%) (p<0.001). Primary revision was necessary in 13 cases (4.7%) due to malreduction of the fracture.

**Conclusion:**

Quality of reduction and functional outcomes following open reduction and internal fixation of displaced two-part surgical neck fractures are related to the surgeon’s experience. In addition, complications and revision rates are less frequent if surgery is conducted by a trauma surgeon performing ≥50 shoulder surgeries per year.

## Introduction

Treatment of displaced proximal humeral fractures can be challenging. Although conservative treatment leads to good results in mildly displaced fractures, outcomes following operative treatment of displaced fractures is heterogeneous. Open reduction and internal fixation is the most frequently performed operative procedure for treatment of displaced proximal humeral fractures [1]. Over the last decade, fracture fixation by use of locking implants has become an established treatment and results have improved in comparison to conventional fixation techniques [2]. However, complication rates following open reduction and internal fixation of displaced proximal humeral fractures still account for up to 30% and numerous studies investigated factors associated with poor outcome [3–6].

Several studies evaluated risk-factors of complications and unsuccessful functional outcomes after locked plating of proximal humeral fractures [7–9]. Comorbidities and the integrity of the medial hinge have been shown to significantly influence clinical outcome and should be assessed for indication preoperatively [7, 10]. Another important factor to avoid complication and achieve a good outcome may be the surgeon itself. Schnetzke et al. have shown, that satisfactory outcome of locked-plate fixation of proximal humeral fractures mainly depends on the quality of fracture reduction. Shoulder function is impaired and complications are more frequently seen, if fractures were reduced nonanatomically [11].

In the treatment of distal radius fractures and hip fractures it has been shown, that quality of fracture reduction and outcomes are related to the experience and practise of the treating surgeon. [12–14]. The role of surgical experience as an independent factor for the quality of reduction in the treatment of proximal humeral fractures remains largely unknown. In addition, the impact of the treating surgeon on functional outcomes and complications following proximal humeral fracture treatment is inadequately understood.

Therefore, the objective of our study was to evaluate the impact of surgeon experience in the treatment of displaced proximal humeral fractures. We hypothesized that in patients undergoing open reduction and internal fixation, fracture reduction would be anatomic and functional outcomes would be excellent, if the surgery was conducted by an experienced surgeon.

## Materials and methods

For this ethical board approved study (LMU No.: 156–12), patients were retrospectively assessed from the institutional proximal humeral fracture database. All data were fully anonymized before evaluation. N = 1,411 Patients with a displaced proximal humeral fracture were treated by open reduction and locking plate fixation between February 2002 and September 2014 (12.5 years). The fracture pattern was determined according to the AO classification [15]. In most cases additional CT-scan was obtained for a comprehensive identification of the exact fracture type. Inclusion criteria were displaced two-part surgical neck type fractures of the proximal humerus (AO 11-A2 and AO 11-A3) treated by locked plating. A total of n = 278 patients were identified and evaluated for this study.

Surgery was conducted in upright beach chair position on a radiolucent table. Every patient received prophylactic intravenous antibiotics as a single-shot and general anaesthesia. Using the deltopectoral approach, surgical reconstruction was achieved in all cases by open reduction and fractures were fixed by use of a locking plate (PHILOS, Synthes DePuy GmbH, Oberdorf, Switzerland). Neither bone grafts nor bone cement or cement augmentation was used to support the fixation in this study. Screws were carefully driven into the subchondral layer, thereby not penetrating the articular surface of the humeral head. To ensure correct screw position and accurate fracture reduction, every step was verified by multi-plane fluoroscopy intraoperatively. When necessary, a screw was repositioned to obtain the intended distance and location of the screw tip relative to the subchondral bone and layer [16, 17].

Surgical experience was determined based on the educational level of the operating surgeon at the time of surgery. With respect to the surgeon’s experience, three subgroups were created:

1. board certified trauma surgeon [TS],
2. senior (>2 years after training) trauma surgeon [STS],
3. board certified trauma surgeon performing ≥50 shoulder surgeries per year [SS], including arthroscopic procedures, fracture treatment around the shoulder and shoulder arthroplasty

Quality of reduction was assessed in each patient from the postoperative true anteroposterior (AP) and outlet-view radiographs of the shoulder conducted within three days from surgery. The AP radiographs were retrospectively analysed by two blinded examiners for quality of fracture reduction (head-shaft displacement, head-shaft alignment) and integrity of the medial calcar hinge. The criteria were adopted from previous studies [10, 18]: A minor varus head-shaft alignment of <120° to 110° was considered to be an acceptable result of fracture reduction, a head-shaft alignment of <110° or >150° was rated as malreduction which is in agreement with previous studies [19–21]. Under these considerations of the quantitative determination of fracture reduction, patients were assigned in 3 groups according to Schnetzke et al. [11]: Overall anatomical fracture reduction, acceptable fracture reduction and malreduced fracture.

True anteroposterior (AP) and outlet-view radiographs of the shoulder were also taken at 6 weeks, 3, 6, and 12 months after surgery to verify the bone healing process and identify variances to the postoperative x-ray and complications. Complications included: Secondary displacement, plate dislocations out of the shaft, screw cut-out, avascular necrosis, infections or hematomas.

At every follow-up examination, patients were also interviewed according to a standardized protocol and physically examined by a member of the orthopaedic surgery staff. The standardized follow-up comprised clinical examinations of the affected shoulder at three, six and 12 months after surgery as well as at final follow-up. Functional outcome measure was the gender- and age-related (normalized) Constant Score [nCS] [22, 23].

### Statistics

Continuous variables were described by median and 95% confidence intervals (95% CI). Descriptive statistics were expressed as mean ± standard deviation. Categorical data were expressed as percentages. Timing of surgery for different fracture patterns was compared using Kruskal-Wallis-Test. Comparisons between normally distributed continuous variables (level of experience) were performed with one-way ANOVA or the t-test and differences among the categorical data were analyzed with chi-square test. Post-hoc analyses for pairwise comparisons were performed using the Bonferroni method for categorical data and the Tukey method for continuous variables. *P* values less than 0.05 were considered statistically significant. Statistical analysis was performed with SPSS version 23 (SPSS Inc., Chicago, IL, USA).

## Results

Between 2002–2014 278 two-part surgical neck type fractures (11-A2 and AO 11-A3) underwent locking-plate osteosynthesis by 22 surgeons. [TS] treated n = 77 (27.7%), [STS] treated n = 116 (41.7%) and [SS] n = 85 (30.6%) patients. Group sizes were significant different in [TS] vs. [SS] p = 0.002 and [STS] vs. [SS] p = 0.004.

**Fig 1:**
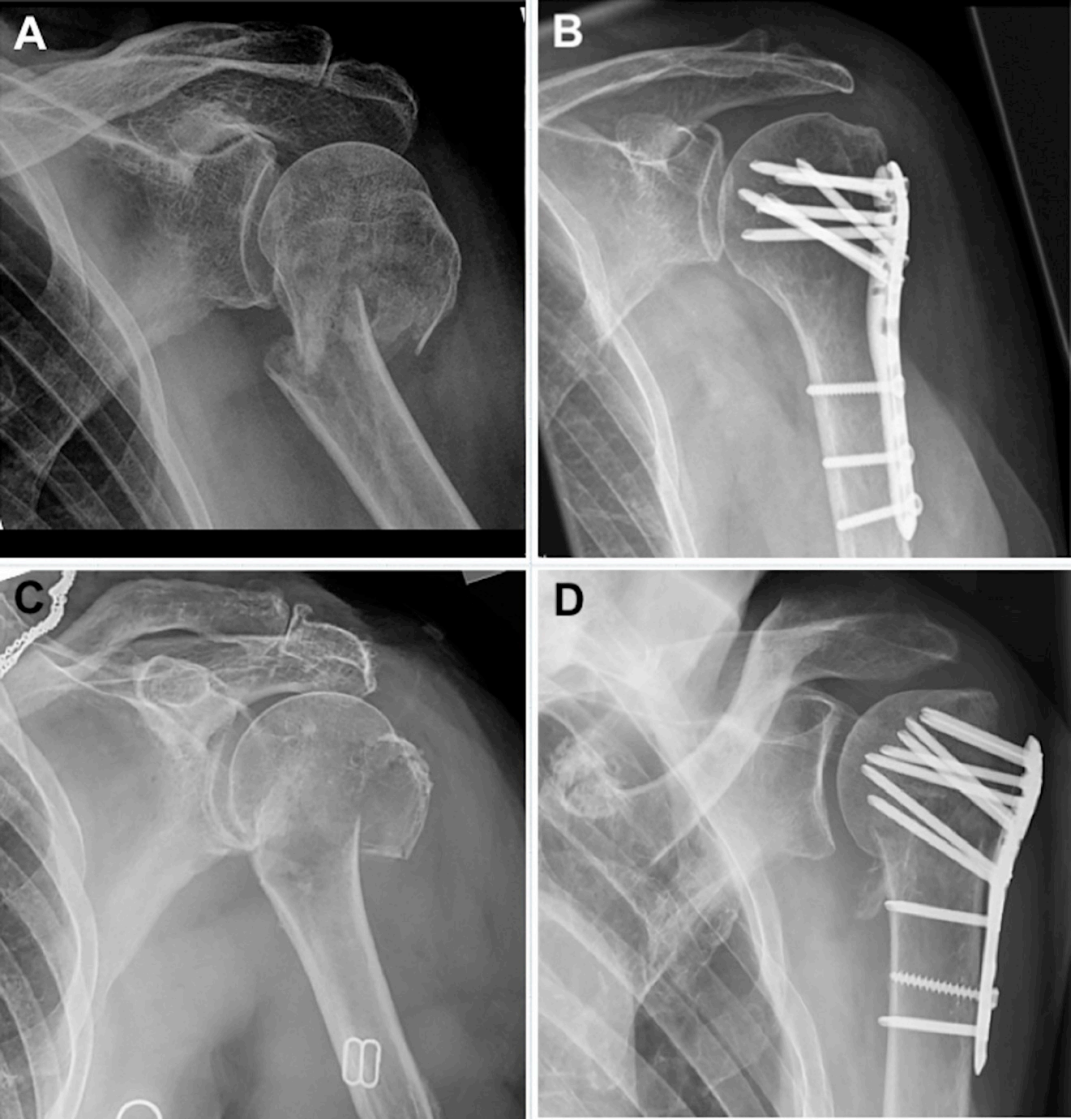
AO 11-A3 fractures before and after ORIF by locking plate. (A)+(B): fracture of a 69 years old female with postoperative anatomical fracture reduction = Inclusion criteria. (C)+(D): fracture of a 72 years old female with postoperative varus malreduction of the humeral head = Exclusion criteria.

**Fig 2:**
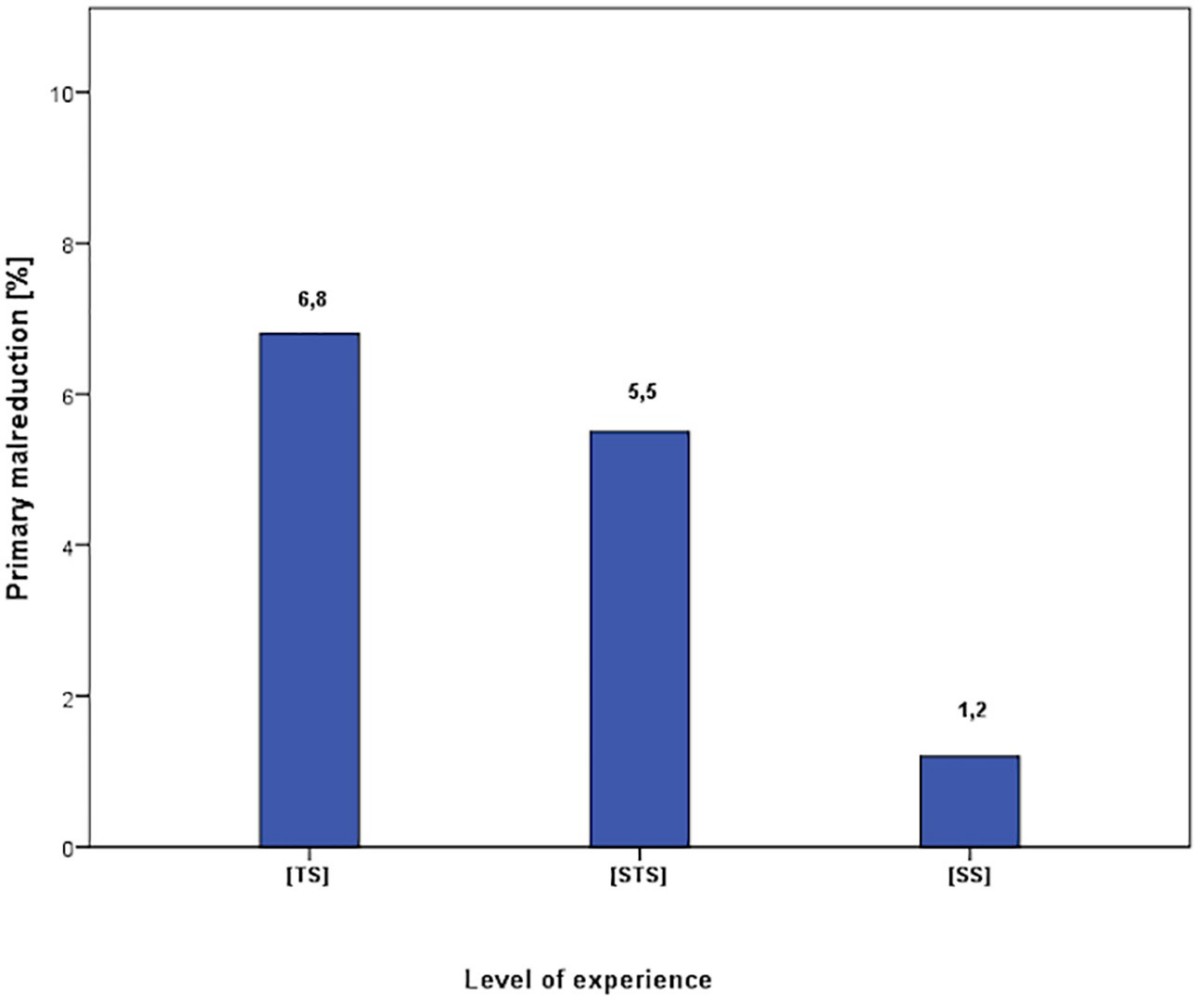
Distribution of primary malreduction and study exclusion rate.

Overall anatomical and acceptable fracture reduction was achieved in n = 265 (95.3%) of the patients. Radiographic evaluation of early postoperative radiographs revealed that n = 248 (93.6%) of the patients had primary anatomic reduction, [TS] 87.9% [STS] 91.2% [SS] 96.5%, p<0.001 [SS] vs. others. In N = 17 (6.4%) cases minor head-shaft alignment was noticed with an overall acceptable reduction. In 13 cases (4.7%) fractures were malreduced and early revision osteosynthesis was performed. Between groups distribution was [TS] 6.8%, [STS] 5.5% and [SS]1.2%, p<0.001 [SS] vs. others. (Figs 1 and 2).

**Fig 3:**
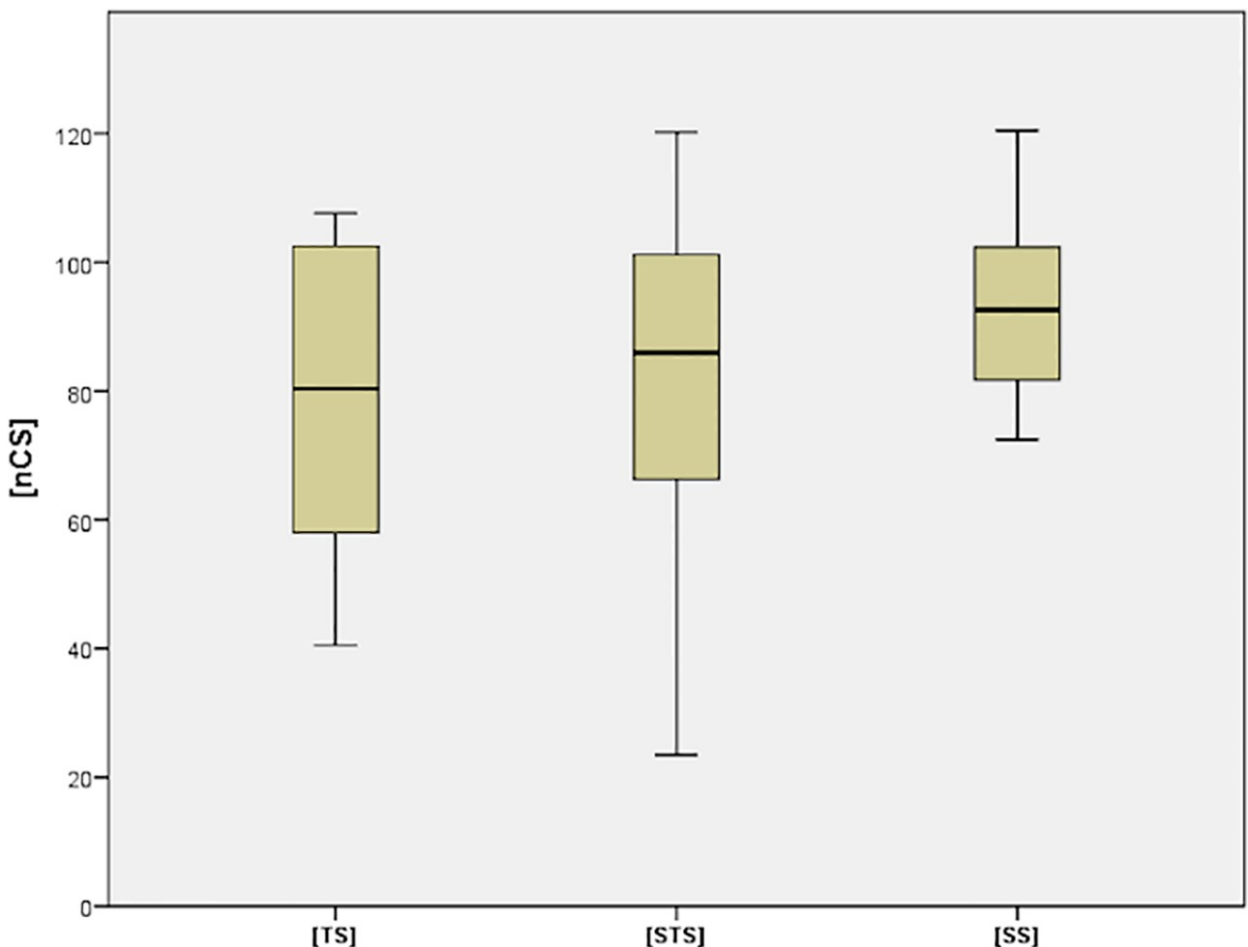
Distribution of the functional outcome (nCS).

Of 265 patients with primary anatomic and overall acceptable fracture reduction, median age at time of surgery was 66.5±17.7 years, 95% Confidence Interval (CI) (65.2,69.5). N = 206 (77.6%) were female (p = 0.78). Distribution of age and gender was not significant between the groups (p = 0.32). The mean functional outcome (nCS) was 85.3±19.6, [TS] 79.6±22.2, [STS] 83.0 ±23.7, [SS] 93.3±13.5, [SS] group vs. others p = 0.01; p = 0.05, [TS] vs. [STS] p = 0.6. (Table 1, Fig 3) Complication resulted in the following distributions [TS] 11.3%, [STS] 11.7%, [SS] 7.8%, [SS] group vs. others p = 0.003; p = 0.01, [TS] vs. [STS] p = 0.83. Complications included loss of fixation with or without screw cut-out in n = 41 cases (15.5%, thereof: 5.8% [TS], 6.8% [STS], 2.2% [SS]). Avascular necrosis n = 4 (1.5%, thereof: 0.7% [TS], 0.7% [STS], 0% [SS]), posttraumatic frozen shoulder in n = 21 cases (7.9%, thereof: 3.2% [TS], 2.9% [STS], 1.4% [SS]) and hematoma in n = 9 cases (3.4%, thereof: 1.8% [TS], 1.4% [STS], 0.3% [SS]). Significance was only found in loss of fixation between [SS] and [STS] (p = 0.04) and [TS] (p = 0.01). There was no case of deep infection or nerve injury. Revision surgery was necessary in [TS] 7.4%, [STS] 8.2%, [SS] 3.9%, [SS], group vs. others p>0.001, [TS] vs. [STS] p = 0.23 (Table 2). We distinguished absolute and relative indications for revision. All of the [TS] revisions were absolutely indicated due to screw cut-out or plate dislocation. In [STS] except for n = 2 (1,7%) moderate secondary displacements all others were absolutely indicated due to relevant secondary displacement, plate dislocations out of the shaft, screw cut-out or infection. All of the [SS] revisions were indicated relatively due to secondary displacement and were discussed critically with the patients. In total 73.7% of all revisions were absolutely indicated. None of the revised patients suffered from peri- or postoperative systemic or other critical harms. Except of n = 3 (15.8%) cases of frozen shoulder after revision surgery, all other patients’ functional outcome benefited from surgery immediately.

**Table 1:** Averaged functional outcome between the three groups nCS [Points]. [CS] was significant superior in [SS] compared to the other groups.

nCS [Points]	p-value
[TS] 79.6±22.2 vs. [STS] 83.0 ±23.7	0.6
[TS] 79.6±22.2 vs. [SS] 93.3±13.5	*0*.*01*
[STS] 83.3 ±25.6 vs. [SS] 93.3±13.5	*0*.*05*

**Table 2:** Averaged functional outcome between the three groups nCS [Points]. [CS] was significant superior in [SS] compared to the other groups.

**Complications [%]**	**p-value**
[TS] 11.3 vs. [STS] 11.7	0.83
[TS] 11.3 vs. [SS] 7.8	*0*.*003*
[STS] 11.7vs. [SS] 7.8	*0*.*01*
**Revisions [%]**	**p-value**
[TS] 7.4 vs. [STS] 8.2	0.23
[TS] 7.4 vs. [SS] 3.9	*>0*.*001*
[STS] 8.2 vs. [SS] 3.9	*>0*.*001*

## Discussion

The main finding of this study is that quality of reduction and functional outcome following open reduction and internal fixation of a two-part surgical neck type proximal humeral fracture is related to the surgeon’s experience. Surgeons with routine experience in shoulder surgeries (>50 cases per year) provide anatomic reduction more frequently, and patients achieve better functional outcomes. In turn, complications are less frequently observed compared to patients treated by surgeons with less experience in shoulder surgery.

In order to discover predictive factors for a successful treatment of proximal humeral fractures, currently, most studies investigate on a variety of patients- and fracture-specific variables i.e. age, bone quality, medial hinge disruption. [12–14]. However, when it comes to open reduction and internal fixation, the surgeon might be another variable of interest. Anatomic reduction has shown to be important in order to facilitate for a good outcome, and complications are less likely if the fracture was reduced anatomically [11]. However, fracture reduction is technically demanding and sometimes every surgeon struggles to achieve so. Gaining more experience in surgical treatment of proximal humeral fractures, thereby increasing the knowledge of typical problems, specific patterns and improving reduction skills, may lead to more accurate operative results. In comparison to distal radial fractures and hip fractures, however, the role of the surgeon’s experience as a potential factor influencing the quality of reduction and the patients’ outcome in proximal humeral fracture treatment was largely unknown, until now. [12–14].

Anatomic reduction was achieved by trauma surgeons [TS] in 87.9% of cases. In comparison, trauma surgeons with >2years experience following their training [STS] achieved anatomic reduction in 91.2%. Thus, more experienced surgeons provided anatomic reduction more often. However, the rate of complications was 11.3% [TS] vs. 11.7%. [STS]. One reason could be, that uncomplicated fracture healing may not only depend on anatomic reduction. Implant position, meticulous screw placement and respect to the periosteal sleeve tissue may also be important factors to avoid complications. Patients showed better functional outcomes if the surgery was conducted by a trauma surgeon performing >50 shoulder surgeries per year [SS]. In comparison, the shoulder function was inferior, when the fracture was treated by a surgeon performing shoulder surgeries less frequently, [SS] 93.3 points normalized Constant-Score vs. [TS] 79.6 points vs. [STS] 83.0 points. Some could argue, that other factors such as management of the long head of biceps tendon and of concomitant rotator cuff tear may influence the functional outcome in a relevant manner as well. However, we may not conclude whether anatomic reduction by itself, or other reasons are responsible for the functional outcome, yet, the functional outcome may be multifactorial. The present study is not able to provide a statement about a minimum number of shoulder surgeries to achieve the results of the [SS] group. Authors’ opinion is not to need a fixed number of ORIF’s of proximal humeral fractures per year, but rather an eye for appearing concomitant injuries as mentioned above. Not only to operate the bone but also to consider all aspects of the injury might be a key factor for better functional outcome.

We evaluated the impact of surgeon’s experience at an educational based university hospital level 1 trauma centre, where surgeons have different experience with fracture treatment of the proximal humerus. For this study, we emphasised on homogeneity in order to reduce potential bias. Although every surgeon has an individual educational training, treatment protocols were institutionally standardized. Thus, we propose, that preoperative diagnostic, the intraoperative setting as well as implants were comparable and patients received the same rehabilitation protocol.

We excluded multisegmental three- and four-part fractures as well as head-split type fractures and fracture dislocation from this study. This was also done for the reasons of comparability. Patients with three- and four-part fracture and head-split type fractures are treated much more individually at our institution, including additive screws, suture cerclage or double plating. Furthermore, patients suffering from a fracture involving the tubercula receive different rehabilitation protocols compared to patients with a two-part fracture of the surgical neck at our institution. We suggest that complex fractures are technically even more demanding and differences between surgeons may be even more distinguishing with respect to their experience. However, surgical neck fractures of the proximal humerus can be highly unstable and are commonly treated by fracture repair.

Besides its retrospective design, this study has several limitations. There was no control group, and a power analysis was not performed. The data are based on a register of surgically treated proximal humeral fractures. A nonoperative control group would be reasonable for further studies. Nevertheless, the study population was larger than that in comparable previous studies and was focused exclusively on proximal humerus fractures involving the surgical neck. Fractures were treated by open reduction and internal fixation with the use of locking plates. Therefore, no information can be given for closed reduction or other fixation techniques. Closed reduction and intramedullary nailing may be advantageous in the treatment of two-part surgical neck type fractures. However, locked plating is an established treatment for such fractures and is performed most frequently.

## Conclusion

Surgeon’s experience has impact on quality of reduction, rate of complication as well as on patient outcome following open reduction and internal fixation of two-part surgical neck type humeral fractures. Outcomes are in favour if the surgery is performed by a trauma surgeon performing >50 shoulder surgeries per year.
